# A whole-food, plant-based program in an African American faith-based population

**DOI:** 10.3389/fnut.2023.1196512

**Published:** 2023-07-14

**Authors:** Faith A. Nyong, Ted D. Barnett, Beth Garver, Maria Dewhirst, Bruce Pollock, Susan M. Friedman

**Affiliations:** ^1^Ascension Mercy, Aurora, IL, United States; ^2^Rochester Lifestyle Medicine Institute, Rochester, NY, United States; ^3^Department of Medicine, University of Rochester, Rochester, NY, United States

**Keywords:** African American, black-, whole food plant-based, faith-based, jumpstart, nutrition, lifestyle

## Abstract

**Background:**

The African American (AA) population is disproportionately impacted by chronic disease as well as many of the leading causes of preventable death, including hypertension, obesity, heart disease, stroke, and type 2 diabetes. In the AA community of Kane County, Illinois, the incidence of chronic disease is particularly high. A standardized Zoom-based group program that gives participants the knowledge, skills, and support to adopt a whole-food plant-based diet has been shown to rapidly improve health. The results of a cohort analysis were analyzed to assess the effectiveness of this program within an AA community characterized by a high burden of chronic illnesses.

**Methods:**

Participants were recruited from a network of 12 AA churches in Illinois to participate in Rochester Lifestyle Medicine Institute’s “15-Day Whole-Food Plant-Based (WFPB) Jumpstart” program. The medically-facilitated 15-Day Jumpstart program provided WFPB nutrition education, coaching, and cooking demonstrations during seven 1 and 2-h Zoom sessions. Participants underwent pre- and post- metabolic screenings to assess for changes in their weight, vital signs, blood sugar, and cholesterol measurements. Changes in diet, biometrics, and patient-centered outcomes from baseline to the end of the program were assessed via paired *t*-tests for the normally distributed measures, and a Wilcoxon signed rank test for measures that were not normally distributed.

**Results:**

Twenty-one AA adults participated. Ten of 16 who provided results had hypertension, 5 had diabetes, 5 had pre-diabetes, and 5 had hyperlipidemia. Participants ate more vegetables (median 2 servings at baseline vs. 3 during the program), greens (1 vs. 3), fruit (2 vs. 3), whole grains (1 vs. 2), and legumes (1 vs. 2). They decreased their consumption of meat, eggs and dairy, added fat, processed foods, and high-fat plant foods (*p* < 0.05 for each comparison). Participants reported significantly better energy (median 5 at baseline vs. 9 during the program, on a 10-point scale), sleep (7 vs. 8.5), and mood (8 vs. 9). Average weight loss was 5.8 pounds (199.9 to 194.1, *p* < 0.001), systolic blood pressure dropped from 129.7 to 119.9 (*p* = 0.02), and total cholesterol dropped from 185.1 to 147.9 (*p* < 0.001). All participants who provided data reported an intent to continue eating at least a partially WFPB diet following the program.

**Conclusion:**

The 15-Day WFPB Jumpstart program led to significant changes in diet, resulting in improvement in several chronic disease measures in this AA community. This rapid improvement can reinforce behavior change. Further large-scale implementation is needed to confirm these preliminary results and to understand whether behaviors and outcomes are sustained.

## Introduction

Chronic disease, exacerbated by obesity, impacts African Americans (AAs) disproportionately (1). The National Health and Nutrition Examination Survey conducted from 2015 to 2016 highlighted that, in comparison to Caucasians, AAs nationwide report a lower intake of fruits, vegetables, dietary fiber, and whole grains, and conversely a higher intake of sugary beverages and processed meats (2). In Kane County, Illinois, 67.8% of adults are overweight or obese (3). In Aurora, the largest city in the county, an AA church community health screening revealed that in 2018, 79% of the women and 86% of the men screened were overweight or obese (4). Additionally, when compared to Caucasians, AAs in Kane County were 3.7 times more likely to be hospitalized for diabetes, were 8.5 times more likely to be hospitalized for hypertension, and were more likely to die from stroke and heart disease than any other race (3). Given that hypertension, diabetes, and heart disease increase the lethality of COVID-19, the pandemic has highlighted the need for a health intervention (5). Poor nutrition plays a significant role in the development of chronic disease, and therefore provides an opportunity to reduce these health disparities (6, 7). Therefore, a health intervention to decrease AA chronic disease and obesity can significantly impact this minority community’s overall health.

African Americans have a distinct cuisine which encompasses a diverse array of food choices and flavors. The African American Heritage Diet maintains that traditional AA cuisine is primarily plant-based, grown from gardens, with substantial quantities of fruits, vegetables, and whole foods (8). However, due to westernized influence and issues of access, current eating patterns are high in salt, oil, high-fat foods, and processed starches, resulting in an overall diet high in sodium, fat, and refined carbohydrates but deficient in fruits, vegetables, and other plant foods (8, 9). African Americans can benefit from adopting a whole-food plant-based (WFPB) diet (10, 11), which has been shown to decrease obesity, diabetes, and heart disease, and to help manage chronic diseases (12). The Rochester Lifestyle Medicine Institute (RLMI) 15-Day WFPB Jumpstart offers individuals the education, structure, and support to understand how to incorporate fruits, vegetables, whole grains, and legumes into their daily eating regimen (11). In this program, participants are instructed to eat only whole-food plant-based options, to limit salt and added sweeteners, and to exclude added oil.

Previous reports of outcomes in a primarily Caucasian population have demonstrated that the Jumpstart program rapidly gives people the knowledge, skills, and experience of a WFPB diet. By doing so, participants realize significant improvements in weight, blood pressure, cholesterol, and blood sugar in 15 days (13). This pilot program was initiated to identify an AA population with a high burden of chronic disease and an interest in following a WFPB diet. The program aimed to evaluate whether they would make significant dietary changes and experience positive health outcomes from moving to such a diet.

The AA participants were recruited from 12 AA churches in Kane County, Illinois. The clergy from the 12 churches were invited to participate in an informational webinar prior to the start of the program. At the webinar, they learned about the prevalence of chronic disease in Kane County AAs and received an overview of the 15-Day WFPB Jumpstart program and its potential benefits for their at-risk parishioners. Afterward, they were asked to distribute program recruitment flyers at their churches.

Overweight and obese parishioners were recruited to participate in the program. Most had at least one other chronic disease indicator. They underwent metabolic testing pre- and post- the 15-Day WFPB Jumpstart program to observe its effects on weight, blood pressure, cholesterol, blood sugar, and mood. The metabolic testing was done at an Ascension community health clinic. The medically-facilitated 15-Day WFPB Jumpstart programming was administered virtually.

## Detail

The 15-Day program components consisted of an informational Zoom session that explained the program structure and the WFPB eating regimen. It also encouraged participants to forward the program information to their medical providers, as incorporating these healthy eating changes could reduce the need for prescription medications. Before beginning the program, the participants had completed a pre-Jumpstart questionnaire regarding their nutrition, the state of their health, and the results of their metabolic testing blood work. During the program, the participants had access to 7 medically-facilitated Zoom-based nutrition education and support sessions.

The program equipped participants with a comprehensive nutrition guide that included shopping lists and recipes for a WFPB diet. Additional nutrition resources were available in a Google Classroom. Between-session communication occurred in the Google Classroom where participants could ask RLMI staff questions about their health, the program, and the WFPB eating pattern. The 15-Day Jumpstart WFPB diet focuses on fruits, vegetables, whole grains and legumes. As has been previously described, “animal products are excluded. High-fat plant foods (e.g., nuts, nut and seed butters, olives, avocado, and coconut) are excluded, as are processed foods and foods with added oil or sugar. Foods that provide a concentrated source of natural sugars, such as dried fruits and maple syrup, are limited to 1 tablespoon per day. Patients are advised to eat 1 tablespoon per day of ground flax seeds to provide omega-3 fatty acids. Patients are not advised to count calories or control portions. In fact, they are encouraged to eat whenever they are hungry—as long as the food is compliant with the program” (13, pp. 375–376).

Participants had access to a cooking and meal preparation demonstration over Zoom. Mid-week check-ins were also held over Zoom. During these check-ins, the participants reported their progress in breakout rooms and asked questions. A virtual potluck allowed participants to share recipes they enjoyed and incorporated into their diets. These recipes were assembled into a cookbook for each participant. At the end of the program, participants completed a post-Jumpstart questionnaire to assess changes to their diet, health, and blood work.

Permission was obtained from the Ascension (formerly AMITA Health) Institutional Review Board to use the data collected from the program participants as part of the QI program. The program was deemed exempt.

One hundred and eight AA adults were invited to participate in the program via email outreach; out of 108 invitees, 21 (19.4%) agreed to participate. The program took place in May and August of 2022. Descriptive statistics were used to characterize the participants at baseline and at the end of the program. Since this was a small pilot study (<20 participants who provided pre-post data), a Shapiro–Wilk test was completed for all the variables, to test for normality. All the biometric measures were normally distributed except for the baseline waist measure. A paired *t*-test was used to evaluate outcomes for the normally distributed measures, and a Wilcoxon signed rank test was used to evaluate the change in waist measure. Several of the dietary components and the self-reported outcomes did not meet the cutoff (*p* < 0.05) for normality. Differences in diet and self-reported outcomes were therefore assessed using the non-parametric Wilcoxon signed rank test. Analysis was completed using IBM SPSS Statistics for Windows, version 29.0 (IBM Corp., Armonk, NY, United States).

Participants ranged in age from 21 to 74, with an average age of 58 (Table 1). 4.8% reported that they were Hispanic, and 4.8% preferred not to say. All but one of the participants were female. Ten of the 16 who provided results about their chronic conditions reported that they had hypertension, 5 had diabetes, 5 had pre-diabetes, and 5 had hyperlipidemia.

**Table 1:** Participant characteristics.

Characteristic (*N* = 21)	
Age—mean (range)	58 (21–74)
Race—African American	100%
Ethnicity	
Hispanic	4.8%
Non-Hispanic	90.5%
Prefer not to say	4.8%
Gender	
Female	95.2%
Male	4.8%

Participants increased their intake of vegetables, greens, fruits, whole grains, and legumes during the Jumpstart program (Table 2). They decreased their intake of meat, eggs and dairy, added fat, processed foods, and high-fat plant foods (Table 2). Average weight loss was 5.8 pounds, systolic blood pressure dropped 9.7 mm Hg, and total cholesterol dropped 37.1 points (Table 3). Participants reported significantly better energy, sleep, and mood (Table 4). All participants who provided data reported an intent to continue eating at least a partially WFPB diet following the program. Five of the 16 who responded said they would eat more WFPB foods; 4 said they would stay on a WFPB diet for the foreseeable future, adding back some high-fat plant foods, and 7 said they would do their best to stay on a WFPB diet but at times might eat some foods that were not part of this eating pattern. Ninety-three percent of the respondents agreed that they had confidence that a very low fat whole-food plant-based diet was the best for their health; 100% stated that they learned important information about the role of nutrition in their health; 100% felt they gained the skills they needed to make positive changes in their health (Table 5).

**Table 2:** Changes in dietary components during the Jumpstart program.

	n	Beginning of program [median (range)]	End of program [median (range)]	Z score	*p-*value**
Measure (servings)					
Vegetables	16	2 (0–3)	3 (1–15)	−3.20	0.001
Leafy greens	16	1 (0–3)	3 (1–6)	−3.46	<0.001
Fruit	16	2 (0–3)	3 (1–6)	−3.11	0.002
Whole grains	16	1 (0–3)	2 (1–6)	−3.10	0.002
Legumes	15	1 (0–3)	2 (1–6)	−3.22	0.001
High fat plant food	16	2 (0–5)	0 (0–2)	−3.08	0.002
Meat	15	3 (0–5)	0 (0–2)	−2.85	0.004
Eggs and dairy	16	1.5 (0–3)	0 (0–1)	−3.02	0.003
Added fat	16	3 (0–10)	0 (0–1)	−3.41	<0.001
Processed food	16	3.5 (0–10)	0 (0–2)	−3.31	<0.001
Rating of adherence*	15	4 (1–9)	9 (6–10)	−3.43	<0.001

*Rated adherence to a very low fat whole-food plant-based diet on a scale from 0 to 10.

**p-value calculated using the Wilcoxon signed rank test.

Results presented only for participants who reported pre-and post-data; total group *N* = 21.

**Table 3:** Jumpstart program biometric measure outcomes.*

	n	Beginning of program [mean (SD)]	End of program [mean (SD)]	Difference [mean (SD)]	95% confidence interval		*P*-value
					Lower	Upper	
Weight (pounds)	17	199.9 (26.3)	194.1 (24.7)	−5.8 (4.1)	−7.9	−3.7	<0.001
Waist circumference (inches)**	15	40.8 (4.3)	38.9 (3.8)	–	–	–	–
Systolic blood pressure (mm Hg)	15	129.7 (9.2)	119.9 (11.4)	−9.7 (13.5)	−17.2	−2.2	0.02
Diastolic blood pressure (mm Hg)	15	77.9 (6.6)	74.5 (6.8)	−3.3 (8.0)	−7.8	1.1	0.13
Cholesterol (mg/dL)	14	185.1 (32.0)	147.9 (28.0)	−37.1 (22.7)	−50.2	−24.0	<0.001
LDL (mg/dL)	15	99.9 (38.3)	79.5 (25.2)	−20.5 (32.0)	−38.2	−2.8	0.03
HDL (mg/dL)	15	57.4 (17.0)	45.7 (15.2)	−11.7 (8.5)	−16.4	−7.0	<0.001
TG (mg/dL)	15	99.1 (47.7)	115.4 (60.5)	16.3 (38.2)	−4.8	37.5	0.12
Glucose (mg/dL)	15	101.8 (16.4)	98.8 (16.4)	−3.0 (10.2)	−8.6	2.6	0.27

*Changes, confidence intervals and *p*-values calculated via paired *t*-test, except for waist circumference.

**Waist circumference was not normally distributed at baseline according to the Shapiro Wilks test; median waist circumference decreased from 39 to 38 inches (Wilcoxon signed rank test, *Z* = −3.31; *p* < 0.001).

Results presented only for participants who reported pre-and post-data; total group *N* = 21.

**Table 4:** Jumpstart program self-reported outcomes.*

	n	Beginning of program [median (range)]	End of program [median (range)]	Z score	*P-*value**
Energy	16	5 (2–10)	9 (7–10)	−3.09	0.002
Sleep	16	7 (2–9)	8.5 (7–10)	−3.21	0.001
Mood	16	8 (2–10)	9 (6–10)	−2.73	0.006
Pain	16	4 (1–8)	4 (1–9)	−1.20	0.23

*Rated on a scale from 0 to 10.

**P-value calculated using the Wilcoxon signed rank test.

Results presented only for participants who reported pre-and post-data; total group *N* = 21.

**Table 5:** Self-report of knowledge, confidence and skills resulting from program (*n* = 15).

	Strongly agree (%)	Agree (%)
“I am confident that I know about the type of eating pattern that is best for my health.”	53.3	40.0
“I learned important information about the role of nutrition in health.”	53.3	46.7
“I gained the skills I need to make changes to my health.”	60.0	40.0

## Discussion

The participants decreased their intake of high fat and sugary foods by focusing on increasing vegetables, leafy greens, fruits, whole grains, and legumes. The session with two cooking demonstrations was pivotal in helping participants understand the “how” of incorporating more plant foods into their diet. During the 2-h virtual session, they were educated on the technique of sautéing vegetables without oil, batch cooking, and meal prep. The session four virtual potluck was instrumental in helping participants understand that eating a whole-food, plant-based diet was not only doable but a delicious way to eat. The virtual potluck required all participants to share their favorite WFPB recipe and a meal photograph, which was incorporated into a participant cookbook. During this session the participants did virtual presentations of their dish, shared preparation tips and answered questions. The virtual, weekly check-in sessions allowed participants to address their barriers to implementing the WFPB diet and assess if they were correctly adhering to the outlined eating regimen. Participants realized that they had preconceived notions on the meaning of eating healthy and that their perceptions did not always correlate with the 15-Day Jumpstart’s literal meaning of fruits, vegetables, whole grains, and legumes. In the 15-Day WFPB Jumpstart, they learned practical and easy-to-implement tips on how to make sustainable lifestyle changes. Because of this education, the participants addressed potential nutritional deficiencies—such as a lack of fiber, and high calorie foods with low nutrient density– that often lead to chronic diseases. In addition, the Rochester staff, due to their willingness to tailor the dietary recommendations to the participants’ cultural preferences, were able to overcome perceived cultural barriers and successfully implement the principles of the 15-Day WFPB Jumpstart in this AA study population.

In addition to improvements in energy, sleep and mood, participants lost weight, had a reduction in abdominal girth, and decreased systolic blood pressure, total cholesterol, LDL and HDL. Except for HDL, all of these changes have been shown to be beneficial to heart health and reducing cardiac risk. Long-term adoption of a very low fat whole-food, plant-based diet has been shown to be effective in reversing heart disease (14). The changes in cholesterol profile seen in this pilot program are consistent with adopting this dietary pattern (13, 15–17). One study of this change in cholesterol profile questioned the predictive value of HDL in populations who do not consume a typical Western dietary pattern, and suggested that, since HDL is part of the assessment for metabolic syndrome, this approach may not be useful in evaluating lifestyle programs that utilize a WFPB dietary pattern (17).

Since some of the 15-Day Jumpstart participants missed multiple sessions, this may have led to less than a full experience of the program. Seventeen of the 21 participants (81%) attended 4 or more sessions, which is the organization’s definition of participation in the program, as it represents at least 5 h of program content, and significant time to interact with program staff. In this population, there are various stressors that may have impacted the participants. Several of the participants were seniors who were primary caregivers to their elementary school-aged grandchildren. Session attendance could have been made more convenient for busy participants by recording sessions for later viewing. It should be noted that sessions should not conflict with church meetings, like mid-week Bible Study or Sunday services, as occurred during this study. For some, in-person meetings tied to these activities might help to boost participation.

Future efforts should focus on motivating the participants to attend all of the sessions and to complete post-Jumpstart questionnaires. Increasing attendance will also give participants more opportunity to develop the skills and obtain the resources needed to make sustainable changes. Faith leaders spend a considerable amount of time attending to the health needs of their parishioners by praying for the sick, visiting patients in the hospital, and planning and conducting funerals. Launching such interventions in faith-based organizations necessitates forming collaborations with clergy and establishing the shared goal of health promotion in their congregations. Clergy can play a vital role by incorporating spirituality into this health intervention (e.g., prayer, scripture reading, devotions, etc.) and can highlight the biblical imperative of pursuing a healthy lifestyle. As a trusted source of information, clergy can encourage participants to regularly attend the sessions. This would lead to a greater likelihood of following the recommendations, which in turn could increase success.

A WFPB diet can be used as a conduit to help AAs lower their chronic disease risk and reduce obesity (18). According to the 2015 National Harris Poll, 8% of AAs adopted a vegan or vegetarian diet as compared to 3% of all other Americans (19). This statistic suggests that the AA community is receptive to eating a WFPB diet. The 15-Day Jumpstart succeeded in providing a framework for how to transition to this dietary pattern, and participants incorporated more fruits, vegetables, and nutrient rich foods into their eating regimen.

Participant recruitment was challenging because many individuals who could have benefitted from this nutritional program did not feel they could implement it. Individuals voiced concerns about eliminating meat and dairy from their diets. They perceived that a WFPB diet would not be satisfying or palatable. A recruitment event that showcased the medical benefits of a WFPB diet coupled with a live cooking demonstration that allowed participants to taste the food can be utilized to increase interest and participation and assuage participants’ concerns.

## Limitations

One of the biggest limitations of the study was its small sample size: 21 participants. However, this was designed as a pilot program to serve as “proof of concept.” Participants made significant changes in eating patterns, and as a result, both experienced improvements in health and expressed an intention to continue at least a partially WFPB diet. A larger, randomized prospective trial would provide more precise estimates of the impact of this program on an African American community. The results seen in this study suggest that a larger study is warranted. Approximately 19% of the individuals who were invited participated in the program, and it is feasible that with increased awareness of the outcomes, the enthusiasm of the participants, and ongoing collaboration with the clergy, the interest in the program could increase.

Not all of the people who signed up participated fully. Some of the program’s sessions had a scheduling conflict with Sunday church services, which created a barrier to regular attendance. Many participants did not thoroughly understand the 15-Day Jumpstart principles which resulted in mistakes in their choices of meal ingredients. This constraint can be addressed by including a component that would evaluate the participants’ understanding of the program’s dietary instructions. For example, including a simple quiz or a hypothetical scenario could confirm that the participants understand the Jumpstart guidelines. This could be done early in the program, to correct any misunderstanding, and enable full participation.

Self-reported outcomes, such as mood, energy, and sleep, were assessed as part of a QI program. As a result, the team used single-question queries rather than more extensive validated tools, in order to maximize response rate. A prospective study using validated tools would help to confirm these preliminary findings. Self-reported measures are subject to reporting bias. Participants are aware of the desired responses, namely, improvement in these measures. However, participants also experienced improvements in objective measures, such as weight, vital signs, and lipids.

## Conclusion

This pilot program was initiated to evaluate whether an AA population with a high burden of chronic disease could be identified, who would be interested in participating in a program to educate them about the benefits of a WFPB diet. Furthermore, it was important to know whether they would make the dietary changes recommended by the program, and whether they would experience positive health outcomes from moving to a WFPB diet.

A nutrition intervention can be utilized to mitigate the disproportionate chronic disease health disparities that plague AAs. This medically-facilitated, 15-Day WFPB Jumpstart program provides the support, education and the practical tools to incorporate increased fruit, vegetable, whole grain and legume intake while eliminating meat, dairy and foods that are processed, contain increased sugar, or are high in fat. This study also highlighted the need to include AA clergy in community health partnerships as they are highly vested in health promotion that benefits their congregants. Their influence as respected faith leaders can be utilized to encourage participation in the 15-Day WFPB Jumpstart program, thereby realizing the shared goal of improving health and decreasing chronic disease.

## Data availability statement

The original contributions presented in the study are included in the article/supplementary material. Further inquiries can be directed to the corresponding author.

## Author contributions

FN assumed multiple responsibilities, including participant recruitment, supervising pre- and post-metabolic screenings, maintaining communication with Frontier journal throughout the submission, peer review, and publication process, ensuring compliance with all specified journal submission guidelines, addressing post-publication correspondence, and serving as the principal investigator. TB was the lead educator for all of the virtual training sessions. BG and MD served as research managers and assisted in conceptualizing and coordinating the health and nutrition intervention in an African American faith-based setting. BP assumed the role of the data coordinator and prepped all data for the statistician. SF was the co-investigator and performed statistical analyses; analyzed and summarized all data; and assisted FN in writing and revising the manuscript. All authors contributed to the article and approved the submitted version.

## Funding

This work was supported by Vegfund (15-Day Jumpstart registration fees) and Ascension Mercy (metabolic fitness testing for each participant). The American College of Lifestyle Medicine paid the access publication fees.

## Conflict of interest

The authors declare that the research was conducted in the absence of any commercial or financial relationships that could be construed as a potential conflict of interest.

## Publisher’s note

All claims expressed in this article are solely those of the authors and do not necessarily represent those of their affiliated organizations, or those of the publisher, the editors and the reviewers. Any product that may be evaluated in this article, or claim that may be made by its manufacturer, is not guaranteed or endorsed by the publisher.
